# “Disk extension beyond the interspace”: an investigation into an alternative nomenclature in diagnostic imaging for displaced canine intervertebral disk material

**DOI:** 10.1186/s12917-015-0421-x

**Published:** 2015-05-14

**Authors:** Lisa K Harder, Davina C Ludwig, Vladimir Galindo-Zamora, Ingo Nolte, Patrick Wefstaedt

**Affiliations:** Small Animal Hospital, University of Veterinary Medicine Hannover, Foundation, Bünteweg 9, D-30559 Hannover, Germany; Small Animal Clinic, Faculty of Veterinary Medicine, National University of Colombia, Carrera 30 45-03 (Ciudad Universitaria), Bogotá, Colombia

## Abstract

**Background:**

Displacement of canine intervertebral disk material can be seen directly in diagnostic imaging modalities such as magnetic resonance imaging and computed tomographic imaging. Canine intervertebral disk herniation can be differentiated into Hansen type 1 and 2 categories by clinical appearance, but anular- and nuclear disk material cannot be distinguished in computed tomographic images. Therefore, we hypothesized that the “Disk extension beyond the interspace”-nomenclature that describes the displacement by the disk contour might aid diagnosis. The aim of this study was to test the reliability of the “Disk extension beyond the interspace”-nomenclature in the evaluation of canine intervertebral disks via magnetic resonance and computed tomographic imaging.

**Results:**

Magnetic resonance and computed tomographic images of 144 intervertebral disks of 43 dogs were evaluated by 3 observers with different degrees of experience from 2 institutions retrospectively. A substantial intraobserver agreement was found, while interobserver agreement was fair to moderate with significant differences in evaluation. Comparison of imaging methods showed a fair to moderate agreement without statistically significant differences in evaluation.

**Conclusions:**

DEBIT-nomenclature cannot be recommended for veterinary clinical usage yet. The largest variability was found in the evaluation of the bulged canine intervertebral disk. The observers’ experience and the imaging method influenced DEBIT- evaluation only slightly, while training and working at different institutions influenced DEBIT-evaluation strongly.

## Background

Displacement of intervertebral disk (IVD) material is common in canine intervertebral disk diseases such as degenerative lumbosacral stenosis and IVD herniation [1-3]. IVD herniation in dogs was divided into type 1 and type 2 herniation by Hansen approximately 60 years ago [4]. Hansen type 1 herniation, also known as disk extrusion, is a displacement of nuclear material through all layers of the annulus fibrosus into the vertebral canal. Hansen type 2 herniation, known as disk protrusion, describes a dorsal displacement of nuclear and annular material into the vertebral canal due to degenerative changes in the annular material. It is not always possible to distinguish between annular and nuclear material in diagnostic imaging. The discrimination of the quality of displaced IVD material when performing computed tomographic (CT) imaging has not been described [5]. Magnetic resonance imaging (MRI) allows the observer to distinguish between annular and nuclear disk material in T2-weighted (T2W) sequences, but only in healthy disks [6,7].

Annular and nuclear material cannot be distinguished in T2W MRI sequences of degenerated IVDs [6,7] due to biochemical changes in the extracellular matrix of the nucleus [8,9]. A lower water content and a shift of proteoglycan composition of the nucleus pulposus results in a lower signal intensity in T2W MRI sequences, making annular and nuclear material iso-intense to each other [7-9]. While IVD degeneration can lead to IVD herniation, herniated intervertebral disks are usually degenerated [2,3].

Additional sequences can help to identify the tissue origin of displaced IVD material. For example, Seiler et al. used a T1-weighted (T1W) sequence to detect annular tears, which enable IVD herniation [10]. Since the origin of intervertebral disk material cannot be seen in each patient and imaging modality, the previously described classification of Hansen does not seem to be the optimal nomenclature in diagnostic imaging. In clinical work, however, Hansen’s nomenclature is somewhat useful for summing up all clinical and imaging results for a patient [11].

Since the dog is a frequently used animal model in studies for human intervertebral disk disease due to comparable degenerative changes and clinical signs of IVD disease [12-14], human medicine may offer a suitable nomenclature to describe displacement of IVD material in diagnostic imaging. The debate in human medicine about nomenclature of displaced IVD material has been under discussion for years. To standardize the multiplicity of terms, the North American Spine Society, American Society of Spine Radiology and American Society of Neuroradiology created recommendations for nomenclature and classification of human disk pathology. These recommendations included the “Disk Extension beyond the Interspace” (DEBIT)-nomenclature [15], which is often used in diagnostic imaging in human medicine [8,16-19]. This morphologic nomenclature states that the physiological position of IVDs is in the interspace between the bony vertebral endplates. Displacement of intervertebral disk material beyond these natural bony limits of the interspace is described by the terms Bulging, Protrusion and Extrusion by the disks contour [17,20]. While disk bulging is a generalized, circumferential symmetrical displacement of IVD material, disk protrusion and extrusion are localized displacements of IVD material [15,17]. Protrusions are defined as broad-based displacements of IVD material, while the diameter of the displaced IVD material of an extrusion is larger than the connection to the parental IVD [18,20].

The origin and quality of IVD material, which are not always clearly visible in veterinary diagnostic imaging, have no influence on the DEBIT-nomenclature. Furthermore, the DEBIT nomenclature is based on the IVD shape, as seen in transverse images that are generated in CT and MR imaging. Consequently the same nomenclature might be suitable for both methods. A standardized nomenclature can facilitate communication among neurologists, surgeons and radiologists in clinical work. Furthermore, a reliable description of dislocated IVD material is important for presurgical planning and definition of the surgical approach.

Parts of DEBIT-nomenclature have been used in studies of canine IVD herniation, but no statistical tests to assess reliability were performed [10,21]. Seiler et al. staged IVD degeneration in low field MRI, defining the term “herniation” as a localized displacement of disk material beyond the limits of the IVD space [10]. Besalti et al. used the term “disk bulging” as a circumferential symmetrical uniform extension of the outer disk margin [21]. Therefore, the aim of this study was to perform DEBIT-classification of displaced canine IVD material in diagnostic imaging, testing reliability and variability. Since MRI and CT are widely used to image displaced IVD material, reliability of the DEBIT-nomenclature was tested in both methods.

## Methods

### Review of imaging data

In total, 43 canine patients of the Small Animal Clinic, University of Veterinary Medicine Hannover Foundation were included in this retrospective study (Table 1). These dogs were presented to the clinic between April 2011 and March 2012 exhibiting signs of pain, reluctance to walk, weakness, lameness and neurological deficits. All dogs were suspected of having spinal cord compression, which was localized by neurological and orthopaedic examinations. Advanced diagnostic imaging, including MRI and CT, was performed by the radiologist on-duty to confirm the diagnosis.

**Table 1:** **Distribution of included breeds**

**Type of breed**	**Breed and number of dogs**	**Number of evaluated disks**
Chondrodystrophic Breeds	Dachshund n = 15	1
		2
		2
		3
		3
		3
		4
		4
		4
		4
		4
		4
		5
		5
		6
	Beagle n = 2	3
		3
	Jack Russell Terrier n = 2	2
		3
	English Cocker Spaniel	3
	Pug	2
	Skye Terrier	1
	Shih Tzu	10
	Bolonka Zwetna	6
Nonchondrodystrophic Breeds	Bernese Mountain Dog n = 2	1
		1
	Dalmatian n = 2	2
		3
	German Shepherd Dog n =2	3
		3
	Berger de Pyrenées	3
	Austrian Black and Tan Hound	4
	German Hunting Terrier	3
	Karelian Bear Dog	2
	Labrador Retriver	3
	Staffordshire Bull Terrier	3
	Hanoverian Scenthound	3
Mix-Breed n = 6		2
		3
		3
		3
		4
		8

The table shows the distribution of included patients in chondrodystrophic and nonchondrodystrophic breeds as well as the number intervertebral disks evaluated of one dog.

MR images were obtained using a 3.0 Tesla high-field MRI-scanner^a^. A spine coil^b^ was used for all examinations. Five dogs with long or multiple areas of interest (length over 70 cm) were examined using the spine coil, and additionally with a neurovascular coil^c^. Images-data of a T2W transverse turbo spinecho sequence (T2W_TSE; echo time 120 ms, repetition time 4.5-12.2 s, slice thickness 1.8-5.0 mm, gap 0.2-0.5 mm) and a T1W multi-Fast-Field-Echo sequence (mFFE; echo time 21 ms, slice thickness 2.2 mm, gap −1.1 - −1.4 mm) with 3 measurements per echo were included in this study.

CT images were obtained with a 64 multislice-detector row CT-scanner^d^. Cervical and thoracic vertebrae were examined with 1.5 mm slice thickness, 120 kV voltage and a current of 200 mAs per slice. The lumbar spine was examined using a 2 mm slice thickness, 140 kV voltage and a current of 200 mAs per slice.

### Evaluation of imaging data

**Figure 1:**
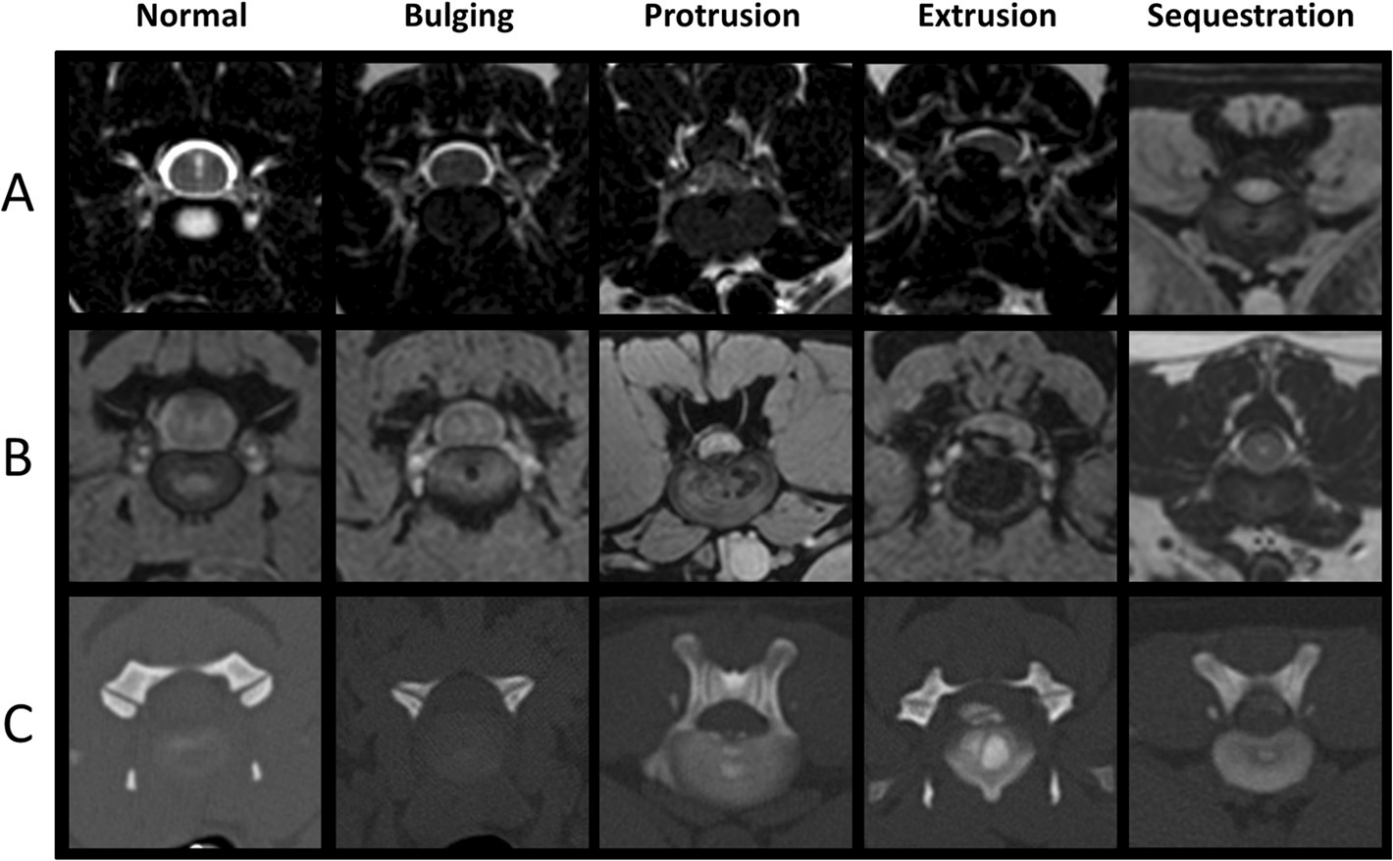
DEBIT in the three imaging methods DEBIT in magnetic resonance and computed tomographic imaging; for each DEBIT category images of the same intervertebral disk are shown in: **(A)**, T2-weighted transverse Turbo Spin Echos sequence; **(B)**, T1-weighted multi-Fast-Field-Echo sequence; **(C)**, transverse computed tomography.

Electronic records of the entire spine were evaluated retrospectively. Image data-sets were included if any IVD was imaged in MRI (T2W_TSE and mFFE sequences) as well as in CT. The IVD did not have to be displaced. In total, 144 IVDs were available for evaluation (Table 1). Electronic records of the transverse MRI sequences and the CT examination were blinded, duplicated and randomized using the pseudo-random function in Statistical Analyses Software (SAS)^e^. Three observers with different degrees of working experience with CT and MRI (observer 1: Two years of experience (LH), observer 2: 5 years of experience (VGZ) and observer 3: 24 years of experience (IN)) evaluated the data sets on a standard computer-screen using the software ImageJ^f^ (Figure 1). The displacement of IVD material was evaluated in transverse data sets of mFFE, T2W_TSE and CT using previously published definitions as follows [15-17,20]: *normal*, no DEBIT; *bulge*, circumferential, symmetrical DEBIT; *protrusion*, focal or asymmetrical DEBIT into the vertebral canal, with the base of the disk being broader than any other diameter of the displaced disk material; *extrusion*, focal DEBIT, the base against the parent disk being narrower than the diameter of displaced disk material (Figure 1). Disk *sequestration* was defined as displaced IVD material with no connection to the disk of origin [15]. All observers were unaware of the clinical findings and diagnosis of the radiologist on-duty.

### Statistical analysis

Weighted kappa analysis was carried out in SAS to evaluate intra- and interobserver agreement in all three diagnostic methods (CT, T2W_TSE, mFFE) and among those methods. Agreement was interpreted according to Landis and Koch as being slight (ĸ 0–0.20), fair (ĸ 0.21-0.4), moderate (ĸ 0.41-0.6), substantial (ĸ 0.61-0.8) and excellent (ĸ 0.81-1) [22]. Bowker’s test was performed to test differences in evaluation among the observers and methods. P-values < 0.05 were considered to be significant.

## Results

43 dogs with a mean age of 6.2 years (5 months-14 years) and a mean weight of 15.9 kilogrammes (3.8-60 kg) were included in this study. A total of 144 intervertebral disks of these patients were available for evaluation including 87 disks of 24 chondrodystrophic dogs, 34 disks of 13 nonchondrodystrophic dogs and 23 disks of 6 mixed-breed dogs (Table 1). The evaluation of magnetic resonance and computed tomographic images by the radiologist on-duty led to the following diagnoses: 36 patients had IVD herniation; 2 had a degenerative lumbosacral stenosis; 3 had myelomalacia without signs of spinal cord compression; 1 had a vertebral fracture due to trauma and 1 showed a massive malformation of the vertebrae resulting in compression of the spinal cord.

### Intraobserver agreement

Intraobserver agreement of DEBIT-classification in T2W_TSE, mFFE and CT (Table 2) was moderate to substantial, with a 95% confidence range of 0.16 to 0.24. One sided p-value to kappa showed a highly significant correlation between two evaluations of the same observer (p <0.0001). P-value by Bowker’s test showed no significant differences in evaluation (p 0.072-0.88) in one observer classifying DEBIT in an MRI sequence or CT.

**Table 2:** **Intraobserver agreement between two evaluations**

**Observer**	**No disagreement**	**Disagreement of 1 classification**	**Disagreement of 2 classifications**	**Disagreement of 3 classification**	**Disagreement of 4 classification**	**κ**	**Limits of confidence**
**T2W_TSE**							
1	110 (76.4)	33 (22.9)	0 (0)	1 (0.7)	0 (0)	0.76	0.68-0.84
2	100 (69.4)	42 (29.2)	1 (0.7)	1 (0.7)	0 (0)	0.61	0.49-0.72
3	95 (66)	33 (22.9)	10 (6.9)	6 (4.2)	0 (0)	0.56	0.44-0.67
**mFFE**							
1	85 (59)	42 (29.2)	16 (11.1)	1 (0.7)	0 (0)	0.54	0.44-0.65
2	113 (78.5)	25 (17.4)	5 (3.47)	1 (0.7)	0 (0)	0.69	0.58-0.8
3	90 (62.5)	44 (30.6)	6 (4,2)	2 (1.4)	2 (1.4)	0.55	0.43-0.66
**CT**							
1	98 (68.1)	39 (27.1)	6 (4.2)	1 (0.7)	0 (0)	0.64	0.54-0.74
2	96 (66.7)	44 (30.6)	3 (2.1)	1 (0.7)	0 (0)	0.63	0.53-0.72
3	82 (56.9)	51 (35.4)	8 (5.6)	3 (2,01)	0 (0)	0.51	0.4-0.62

Intraobserver agreement between two evaluation session for determination of DEBIT-nomenclature in 144 intervertebral disks obtained from 43 dogs.

### Interobserver agreement

**Figure 2:**
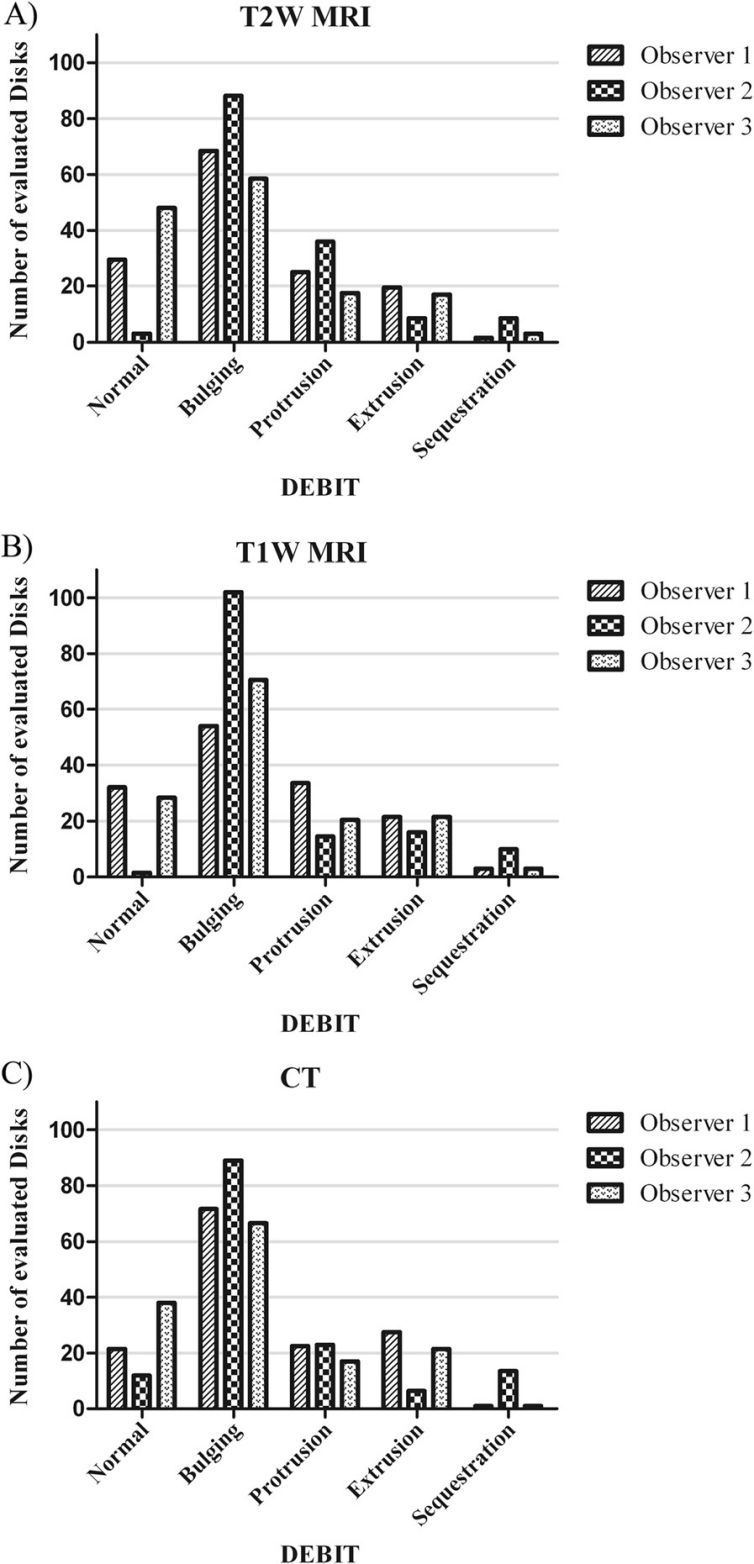
Evaluation of DEBIT of all observers in MRI and CTThe bars show the number of intervertebral disks that were evaluted as being normal, bulged, protruded, extruded and sequestrated. Each bar is the mean value of two classification sessions of one observer. **A)**: Classification of DEBIT using T2W_TSE images; **B)**: Classifiacation of DEBIT using mFFE images; **C)**: Classification of DEBIT using CT images.

Interobserver agreement (Table 3) was moderate, despite a fair agreement between observer 2 and 3 in the evaluation of DEBIT in T2W_TSE. The 95% confidence range was small with 0.18 to 0.22 and one-sided p-value to kappa (p <0.0001) showed a high correlation. Statistically significant differences in evaluation could be found between observer 2 and 3 for the evaluation of T2W_TSE sequences (p <0.0001); as well as between observer 1 and 2, and between observer 2 and 3 in the case of the mFFE sequence (p <0.0001). In CT, statistically significant differences in evaluation were seen between observer 1 and 2 (p 0.024) as well as between observer 2 and 3 (p 0.044). Those differences in evaluation can be seen in Figure 2.

**Table 3:** **Interobserver agreement in DEBIT-nomenclature**

	**Kappa**	**Limits of confidence**	**One-sided p-value to κ**	**p-value of Bowker’s test**
**T2W_TSE**				
1&2	0.5	0.41-0.59	<0.0001***	0.39
1&3	0.59	0.49-0.69	<0.0001***	0.35
2&3	0.37	0.28-0.49	<0.0001***	<0.0001***
**mFFE**				
1&2	0.43	0.33-0.53	<0.0001***	<0.0001***
1&3	0.58	0.47-0.69	<0.0001***	0.51
2&3	0.48	0.38-0.58	<0.0001***	<0.0001***
**CT**				
1&2	0.42	0.32-0.52	<0.0001***	0.024*
1&3	0.51	0.4-0.62	<0.0001***	0.49
2&3	0.41	0.31-0.51	<0.0001***	0.044*

One-sided p-value to κ shows statistical significant agreement between the two evaluations at: *p < 0.05, ***p < 0.001; Bowker’s test shows significant differences in evaluation of DEBIT by two observers with p-value: *p < 0.05, ***p < 0.001.

### Comparison between methods

Comparing the results of DEBIT-evaluation between the different methods, a moderate agreement was found between the evaluation of T2W_TSE and mFFE images (Table 4). Comparing the evaluation of both MRI sequences with the results of evaluation of CT images, a fair to moderate agreement was found. A significant correlation was found among all methods (p < 0.0001). No significant differences in evaluation among all three methods were found in Bowker’s test (p 0.1-0.84).

**Table 4:** **Comparison of DEBIT-nomenclature in different imaging methods**

	**Kappa**	**Limits of confidence**	**One-sided p-value to κ**	**p-value of Bowker’s test**
**T2W_TSE:mFFE**				
1	0.5	0.38-0.62	<0.0001***	0.67
2	0.56	0.45-0.67	<0.0001***	0.16
3	0.45	0.33-0.56	<0.0001***	0.59
**T2W_TSE:CT**				
1	0.47	0.35-0.59	<0.0001***	0.67
2	0.51	0.39-0.64	<0.0001***	0.5
3	0.36	0.24-0.48	<0.0001***	0.84
**mFFE:CT**				
1	0.4	0.28-0.52	<0.0001***	0.12
2	0.5	0.33-0.58	<0.0001***	0.1
3	0.39	0.27-0.51	<0.0001***	0.61

One-sided p-value to κ shows statistical significant agreement between the evaluation of DEBIT by one observer in two different imaging methods: ***p < 0.001. Bowker’s test shows significant differences in evaluation of DEBIT by one observer in two different imaging methods with p-value: ***p < 0.001.

The results of the evaluation are summarized in Figure 2, which shows the evaluation of DEBIT of the three observers in all methods. For each observer and method the mean value of two classification sessions was calculated. More normal IVDs were identified in the evaluations by observers 1 and 3 in all methods than in the evaluations by observer 2. Observer 2 evaluated more IVDs as being bulged in all methods than observers 1 and 3. Evaluation of disk protrusion by observer 3 showed fewer protruded IVDs than observer 1 in all methods. These two observers found approximately the same number of protruded disks in T2W_TSE and CT evaluation, while they scored more IVDs as being protruded in mFFE. Observer 2 had different tendencies in the three methods, evaluating more IVDs as being protruded in T2W_TSE than in CT, followed by mFFE. Fewer IVDs were judged as extruded by observer 2 compared with observers 1 and 3, while observer 2 judged more IVDs as being sequestrated than observers 1 and 3.

### Interpretation of the term sequestration

In the proposed nomenclature, the term sequestration describes displaced disk material that has no contact to the IVD. Interpretation and usage of the term “no connection to the disk of origin” was different among the observers. The results revealed that observers 1 and 3 used the term sequestration for displaced IVD material lying in the vertebral canal, but not directly dorsal to the intervertebral disk space. They used the term extrusion for displaced IVD material that was positioned dorsal to the intervertebral space, even if the connection could not be seen clearly. Observer 2 used the term sequestration for displaced IVD material that was dorsal to the intervertebral disk space and showed no obvious contact zone.

## Discussion

The aim of this study was to test the reliability of DEBIT-nomenclature in the evaluation of canine IVD displacement using different imaging methods. A good consistency in usage of a nomenclature is important, especially in preoperative planning when information about the current state of the IVD has to be communicated from one person to another. The consistent description of displaced IVD material, even by different investigators, is crucial for the best possible surgical approach or intervention. While good intraobserver reliability was found for each of the evaluated modalities (Table 2), interobserver agreement showed mainly moderate results, with statistically significant differences in evaluation of DEBIT between observers 2 and 3 (Table 3). It can be concluded that intraobserver reliability is said to be better than interobserver reliability, as has been found in another study [23]. The wide variability in evaluation showed that DEBIT-nomenclature cannot yet be recommended for evaluation of canine IVD displacement. Four main factors were found in literature that influence the variability of the examination: the imaging method, the observers’ experience, the observers’ institution and the classification system used [17,24]. The potential influence of these four factors on the variability of DEBIT-nomenclature in the present study is considered below.

Regarding the imaging method, similar results were seen in the evaluation of DEBIT in CT and MRI (Tables 2 and 4). MRI is the gold standard method for diagnosing displaced disk material causing spinal cord compression [11,25]. The intervertebral disk, the spinal cord and the cerebrospinal fluid are directly visible in a T2W MRI sequence [11,25,26]. An additional T1W sequence is a useful tool for imaging annular tears and separate subdural haemorrhages from displaced IVD material [10,25]. The present study showed that both sequences used separately allow a comparable classification of displaced IVD material without statistically significant differences in evaluation.

The comparison of evaluation of MRI with CT showed a slightly lower reliability but no statistically significant differences in evaluation (Table 4). Noncontrast CT shows the spinal cord surrounded by the epidural fat, which has an intermediate attenuation [5,25]. The intervertebral disks are isodense to the long back muscles or hyperdense due to calcification. Displaced IVD material can be identified as a hyperattenuating mass in the vertebral canal if it is calcified [5,11]. In the present study 62 IVDs showed calcified areas, mainly in the nucleus pulposus, but displaced IVD material was calcified in 24 cases only. If the displaced disk material is not calcified, it cannot be clearly seen and instead a loss of epidural fat or displacement of the spinal cord may help to identify the location of displaced IVD material [5,27].

The present study highlights that canine displaced IVD material can be seen in CT images without need for CT-myelography due to statistically significant similar results of DEBIT-evaluation in noncontrast CT compared to MRI. Nevertheless the comparison of the reliability of DEBIT-nomenclature in CT and MRI is limited by the other variables like the observers’ experience and institution.

In the present study, observers had different degrees of experience in diagnostic imaging. The evaluation of displaced IVD using image data obtained from different modalities showed that experienced observers (observers 2 and 3) had similar results in intraobserver agreement in all methods (Table 3). The less experienced observer (observer 1) had a smaller intraobserver agreement in the evaluation of displaced IVD material in mFFE than in the other sequence and CT. This finding can be explained by the circumstance that the observer had only little experience with that particular sequence.

The experienced observers (2 and 3) were familiar with the evaluation of structural changes in the spinal cord in mFFE sequence. However, the evaluation of the IVD contour in mFFE sequence was a new task for all three observers. Due to a lack of experience with the mFFE sequence in general, the evaluation of DEBIT in that sequence might have been more challenging for observer 1 when compared to the evaluation of the T2W_TSE. Surprisingly, a moderate interobserver agreement was found between the observer with less experience and the two more experienced observers in the present study when considering all modalities. In the present study, observers 1 and 3 worked at the same institution, while observer 2 worked at another institution. The statistically significant differences in evaluation of DEBIT in observer 2 compared to observer 1 and 3 (Table 3, Figure 2) indicate that training and working in different institutions influences the evaluation of displaced IVD material markedly. Working at different institutions had a greater influence on evaluation than observers’ experience in the present study, probably because differences in training influence the classification accuracy of the nomenclature used in this study.

Since the DEBIT-classification system does not have a continuous scale, the cut-off point between two categories is somewhat subjective. Thus, each observer independently defines his or her own cut-off point between two classifications. In all methods, differences in evaluation were seen between the normal and the bulging IVD (Figure 2). Observers 1 and 3 defined more IVDs as being normal than observer 2. Generally, disk bulging is a term which is not often used in veterinary practice, although disk bulging may lead to pain by activating nociceptive innervation due to stretching dorsal anular fibers or the dorsal longitudinal ligament [11]. Accordingly, all observers dealt with a new term, which had not been discussed previously in their training. That might be one reason for the differences in evaluation. In human medicine, the largest differences in evaluation were seen between the normal and the bulging IVD evaluated by two experienced observers working at the same institution; hence, our results agreed with those of the human studies [17,20].

Variability was found when evaluating disk bulging and disk protrusion (Figure 2). Differences in evaluation can result from the choice of image slices for evaluation. An IVD can show a symmetrical extension on one slice whereas on the next image slice a slight, focal rise of annular material may be seen, which can be interpreted as disk protrusion. In the present study image stacks were available for evaluation, so the observer could choose the image used for evaluation. That might have increased the variability in evaluation of disk bulging with respect to disk protrusion, as previously described. Discrepancies in evaluation of the bulged versus the protruded human IVDs were also seen in the studies of Brant-Zawadzki et al. and Milette et al. [17,20]. In both studies the evaluation was performed by experienced observers working at the same institution, so an increased variability between the bulged and protruded IVD does not seem to depend on the observers’ experience.

The different interpretation of the term sequestration by the observers most likely had a negative influence on the reliability of the DEBIT-nomenclature. Using the term sequestration with a different meaning may lead to different results among users. Obviously, the definition of the term disk sequestration was not sufficiently precise. Thus an exact predefinition of mentioned term is required in future Application of DEBIT- nomenclature. We suggest to use the term sequestration for displaced disk tissue without connection to the parental disk, and which is lying not dorsally of the intervertebral disk space.

The most important limitation of the study was the usage of transverse image slices only. Although a reconstruction of sagittal- and dorsal-plane slices was possible in the imaging software used, the slice thickness of the transverse stacks was not suitable for reconstruction. Having additional image planes might increase the reliability of the evaluation.

In the present study, Bowker’s test showed statistically significant differences in evaluation of DEBIT-nomenclature between different observers. Therefore, consistency concerning the terms disk protrusion and extrusion was too small to compare DEBIT-nomenclature with Hansen’s nomenclature. Due to the fact that most canine intervertebral disk herniations can be clinically divided into Hansen Types 1 and 2 [11,28], further studies with a smaller variation in usage of DEBIT-nomenclature are needed to test whether it can be integrated into the nomenclature proposed by Hansen or vice versa.

## Conclusions

DEBIT-nomenclature showed an only moderate reliability in classification of canine displaced IVD material within and between both imaging modalities. Therefore, it cannot be recommended for clinical usage yet. The term “disk bulging” was not routinely used by any of the observers. Consequently, discrimination between disk bulging and the normal respectively the protruded IVD showed the largest variability. Since similar results were found in human medicine, regular practical experience will lower that variability [17,20,24]. The term “sequestration” was differently interpreted by the observers working at different institutions. Therefore a more appropriate definition has to be found for this term. The obvious influence of the observers’ institution on the variability of DEBIT-nomenclature underlines the challenge in introducing a new nomenclature into clinical practice.

## Endnotes

^a^Achieva 3.0 T TX, Philips Medical Systems, Best, Netherlands.

^b^SENSE Spine Coil 3.0 T, 15 Channel, Philips Medical Systems, Best, Netherlands.

^c^SENSE NV Coil 3,0 T, 16 Channel, Philips Medical Systems, Best, Netherlands.

^d^Brilliance 64, Philips Medical Systems, Best, Netherlands.

^e^Statistical Analysis Software, SAS Institute Inc., Cary, North Carolina, USA.

^f^Image Processing Analysis in Java, National Insitute of Health, Bethesda, USA.

## Abbreviations

CT: Computed tomography
DEBIT: Disk extension beyond the interspace
IVD: Intervertebral disk
MRI: Magnetic resonance imaging
mFFE: multi fast field echo
T1W: T1-weighted
T2W: T2-weighted
T2W_TSE: T2-weighted turbo spin echo
